# A geometric analysis of hallux valgus: correlation with clinical assessment of severity

**DOI:** 10.1186/1757-1146-2-15

**Published:** 2009-05-14

**Authors:** Carlos Piqué-Vidal, Joan Vila

**Affiliations:** 1 Centro Médico Teknon, Barcelona, Spain; 2 Grupo de Epidemiología y Genética Cardiovascular (EGEC-ULEC), Institut Municipal d'Investigació Mèdica (IMIM-Hospital del Mar), CIBER Epidemiología y Salud Pública (CIBERESP), Barcelona, Spain

## Abstract

**Background:**

Application of plane geometry to the study of bunion deformity may represent an interesting and novel approach in the research field of hallux valgus. For the purpose of contributing to development of a different perspective in the assessment of hallux valgus, this study was conducted with three objectives: a) to determine the position on the intersection point of the perpendicular bisectors of the longitudinal axes of the first metatarsal and proximal phalanx (IP), b) to correlate the location of this point with hallux valgus deformity according to angular measurements and according to visual assessment of the severity carried out by three independent observers, and c) to assess whether this IP correlated with the radius of the first metatarsophalangeal arc circumference.

**Methods:**

Measurements evaluated were intermetatarsal angle (IMA), hallux valgus angle (HVA), and proximal phalangeal articular angle (PPAA). The Autocad^® ^program computed the location of the IP inside or outside of the foot. Three independent observers rated the severity of hallux valgus in photographs using a 100-mm visual analogue scale (VAS).

**Results:**

Measurements of all angles except PPAA showed significantly lower values when the IP was located out of the foot more distantly and vice versa, significantly higher values for severe deformities in which the IP was found inside the foot (*p *< 0.001). The IP correlated significantly with VAS scores and with the length of the radius of the circle that included the first metatarsophalangeal arc circumference (*p *< 0.001)

**Conclusion:**

The IP is a useful indicator of hallux valgus deformity because correlated significantly with IMA and HVA measurements, VAS scores obtained by visual inspection of the degree of deformity, and location of the center of the first metatarsophalangeal arc circumference.

## Background

Different radiographic measurements are widely used to assess angular deformity in patients with hallux valgus. Conventional measures of severity of hallux valgus including the hallux valgus angle (HVA) and the first intermetatarsal angle (IMA) are well accepted and integrated universally in clinical practice and surgical decision making. Severity of each parameter is based of radiographic cut-off points [1-17]. The importance and validity of the distal metatarsal angle (DMAA) and the proximal phalangeal articular angle (PPAA) is controversial [14,18,19]. Other variables (e.g. position of the sesamoids, articular congruence, range of motion testing, first ray mobility measurement, level of osteoarthritic change within the first metatarsophalangeal joint, etc.) may be assessed for presurgical planning purposes.

Visual inspection of foot has been described as a screening method for hallux valgus in children [20]. Moreover, a non-invasive clinical assessment tool (the Manchester scale), consisting of four standardized photographs, has been shown to provide a valid representation of the degree of hallux valgus deformity determined from radiographic measurement HVA and IMA [21,22]. This instrument is a simple, non-invasive screening tool for clinical and research purposes. In contrast, recent technological advances now allow the radiographs to be digitalized, measured with computer tools (e.g. AutoCAD^® ^software program), stored electronically, and retrieved with a computer. Computer-assisted analysis of skeletal radiographs is increasingly introduced in the field of hallux valgus [23-29].

In a previous study based on digitized images of angular measurements, the position of the center of a circle formed by the first metatarsophalangeal arc circumference correlated significantly with HVA, DMAA, and IMA measurements [29]. The circle's center location was associated with different degrees of hallux valgus deformity. Although this single point integrating different angular measurements represents a new investigational approach to study the severity of hallux valgus, drawing a circumference manually on radiographs is difficult and time consuming in clinical practice. However, application of this research model to the intersection point of the perpendicular bisectors of the mid axes of the first metatarsal shaft and the first proximal phalanx (IP) may have more practical relevance since these lines can be easily drawn on weightbearing radiographs.

Application of plane geometry to the study of bunion deformity may represent an interesting and novel approach in the research field of hallux valgus. For the purpose of contributing to development of a different perspective in the assessment of hallux valgus and based on previous findings of the correlation of the first metatarsophalangeal circumference with angular measurements [29], we here studied the position of the IP to assess whether there was a correlation between this point and (a) the degree of hallux valgus deformity according to angular measurements, (b) the severity of hallux valgus assessed by three independent observers using a visual analogue scale (VAS), and (c) the center of the circle of the first metatarsophalangeal arc circumference [29].

## Methods

All consecutive patients with hallux valgus evaluated roentgenographically during the preoperative workup studies over 1-year period (from January 2005 to January 2006) were included in a cross-sectional study. A control group of normal feet was also included. The control group was included patients without hallux valgus who were visited because of other orthopaedic conditions and agreed to participate in the study. Patients with hallux valgus and controls gave written consent to undergo the study procedures. Exclusion criteria included previous foot surgery and neurological diseases.

### Angular measurements

Dorsoplantar radiographs for weightbearing conditions were performed with the patients standing on both feet with the knee extended. The medial border of the foot was aligned to avoid internal or external rotation of the leg. The foot was pointed straight forward in neutral rotation, parallel to the medial sagittal plane. The X-ray beam was inclined 15° in an anterior-posterior direction centered on the second tarsometatarsal joint at a distance of 100 cm. Radiographs were photographed with a digital camera (Canon^®^S40).

Measurements evaluated were HVA, IMA and PPAA. HVA formed by the intersection of the longitudinal axes of the first metatarsal and the proximal phalanx and IMA formed by the intersection of the longitudinal axes of the first and second metatarsals were measured using mid diaphyseal reference points [30]. The PPAA is the angle subtended by a line drawn perpendicular to the phalangeal articular surface and the longitudinal axis of the proximal phalanx [31]. All measurements were performed by an independent observer who was blinded to the patient's medical record and unaware of the purpose of the study, using an AutoCAD 2000^® ^(Autodesk Inc., San Rafael, California, USA). The HVA was categorized as normal (< 15°), mild (15–20°), moderate (21–39°), and severe (≥ 40°); the IMA as normal (< 9°), mild (9–11°), moderate (12–17°), and severe (≥ 18°); and the PPAA as normal (< 6°), mild (6–10°), moderate (11–20°), and severe (≥ 21°).

### Perpendicular bisectors of the longitudinal axes of the first metatarsal and proximal phalanx

**Figure 1:**
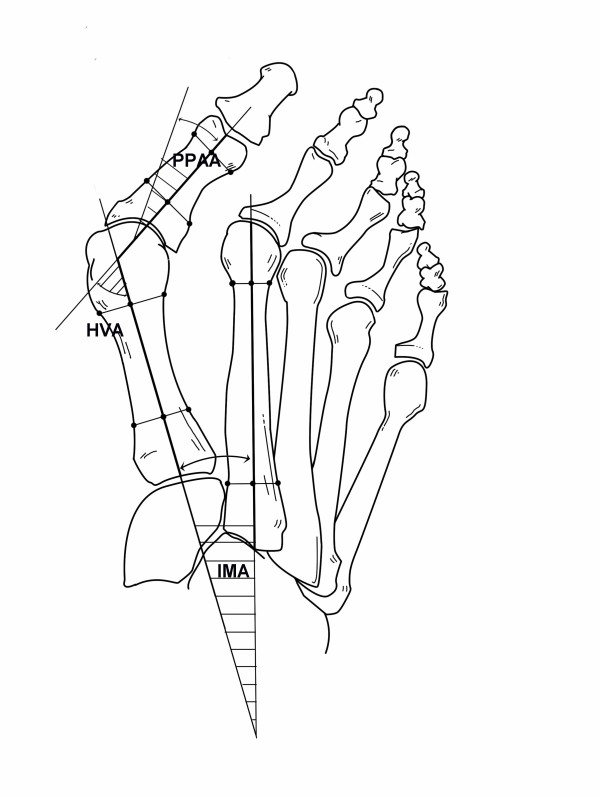
**Measurements of the hallux valgus angle (HVA), the first intermetatarsal angle (IMA), and the proximal phalangeal articular angle (PPAA)**.

**Figure 2:**
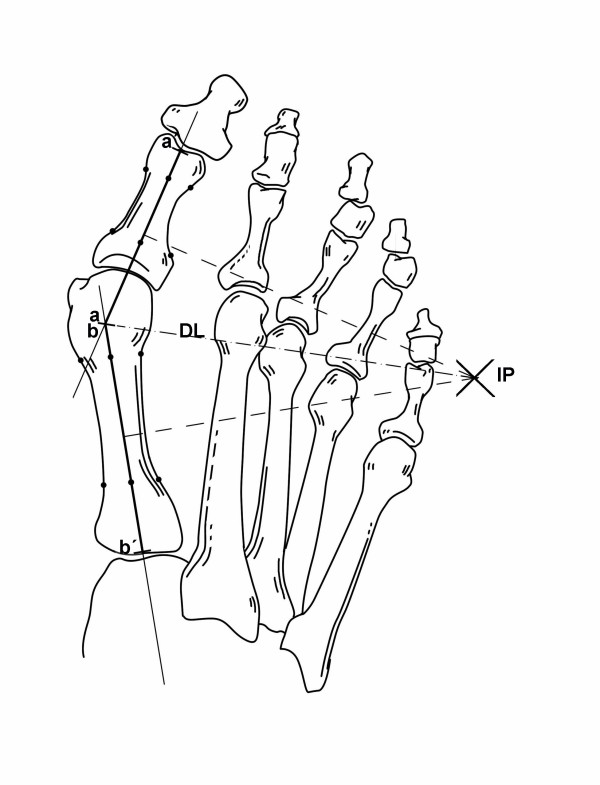
**Position on the intersection point (IP) of the perpendicular bisectors of the longitudinal axes of the first metatarsal shaft (line segment a-a') and proximal phalanx (line segment b-b')**. Distance length (DL) is the line segment from IP to a/b.

After angular measurements had been taken (Figure 1), perpendicular bisectors of the longitudinal axes of the first metatarsal and proximal phalanx were drawn (Figure 2). The segment for the first metatarsal had the starting point in the intersection of the longitudinal metatarsal axis with the line of the base of the first metatarsal, and the end point in the intersection with the prolongation of the longitudinal axis of the first phalanx of the big toe. The other segment had the starting point in the intersection of the phalangeal and metatarsal axes and the end point in the intersection of the phalangeal axis with the distal end of the phalanx.

The Autocad^® ^program computed the location of the IP and the length of a line segment (named 'DL' = distance length) joining the IP and the longitudinal metatarsal and phalangeal axes. Two categories were established for the site of the IP: inside the foot and outside the foot. Width of the foot was measured radiographically as the distance between the maximal prominence of the fifth and first metatarsals.

### First metatarsophalangeal arc circumference

**Figure 3:**
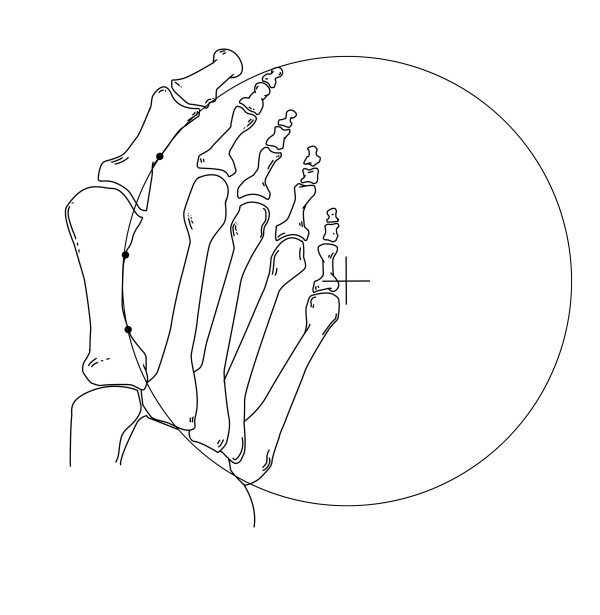
**Position of the center of the circle formed by the first metatarsophalangeal arc circumference**.

The first metatarsophalangeal arc was defined by the midpoint of the curvature of the first phalanx and by placing two points each at the proximal and distal metadiaphyseal metatarsal junctions at the lateral borders (Figure 3). Details of drawing of the circle have been previously reported.^29^

### Clinical assessment of hallux valgus severity

**Figure 4:**
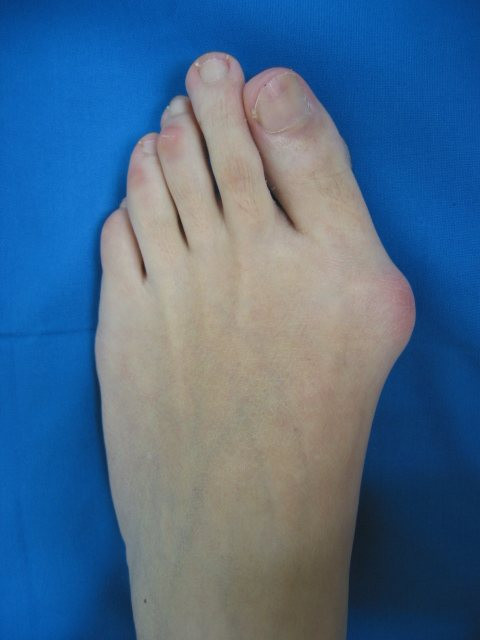
**Representative image of hallux valgus deformity**.

After radiographs had been taken and with the patient in the same position, a macroscopic photograph of the involved forefoot was taken using the same digital camera (Canon^®^S40). For this weightbearing view, the patient stands with their toes pointing straight ahead, knees fully extended, and weight distributed evenly on both feet. The medial border of the foot was aligned to avoid internal or external rotation of the leg. The camera was strictly positioned perpendicularly to the bearing surface of the foot, for which a tripod and a spirit level were used. The distance was not standardized; for each case the distance necessary so that the foot occupied the entire screen area was selected. Each foot was photographed separately. A representative image of a clinical photograph is shown in Figure 4. Three independent observers rated the severity of hallux valgus in forefoot photographs using a visual analogue scale (VAS) The VAS consisted of a 100 mm horizontal line, the left end representing 'normal appearance or absence of hallux valgus deformity' (0 mm) and the right 'maximum hallux valgus deformity' (100 mm). Each observer was unaware of the purpose of the study and blinded regarding other observers' assessments.

Institutional review approval was given for this study.

### Statistical analysis

The normal distribution assumption of continuous variables was assessed by normal probability plots. The Student's *t *test was used to compare means differences among groups. When more than two groups were compared a one-way analysis of variance (ANOVA) was used, correcting the *P *value for multiple comparisons by Tukey's method. The relationship between continuous variable was assessed by the Pearson's product-moment correlation coefficient. A single measure of VAS scores (summary VAS score or s-VAS) was obtained from VAS scores of the three observers. It was done by a factor analysis with a varimax rotation. To assess the influence of angular measurements on DL, a multivariate lineal regression analysis was performed. Statistical significance was set at *p *< 0.05. The Statistical Analysis System (SAS) statistical software package (version 8.0) (SAS Institute, Cary, North Carolina, USA) was used for the analysis of data.

## Results

A total of 301 dorsoplantar weightbearing radiographs from 176 patients with hallux valgus (bilateral hallux valgus, n = 90; unilateral hallux valgus, n = 86) and 35 controls were analyzed. There were 192 women and 19 men, with a mean age of 56 ± 14 years (range 28–87 years). The classification of hallux valgus deformities based on angular measurements is shown in Table 1. Angular values showed a mean (± standard deviation, SD) values of 30.3 (12.6)° for HVA, 12.3 (3.8)° for IMA, and 4.5 (4.9)° for PPAA. Angular values in controls were as follows: 10.6 (3.2)° for HVA, 8.2 (2.2)° for IMA, and 7.5 (4.0)° for PPAA.

**Table 1:** Classification of severity of hallux valgus deformities according to angular measurements in 301 radiographs

Angle	Severity	No. cases	Percent
HVA	Normal, < 15°	35	12
	Mild, 15–20°	42	13
	Moderate 21–39°	153	51
	Severe, ≥ 40°	71	24
			
IMA	Normal, < 9°	58	19
	Mild, 9–11°	76	25
	Moderate, 12–17°	135	45
	Severe, ≥ 18°	32	11
			
PPAA	Normal, < 6°	116	38
	Mild, 6–10°	115	38
	Moderate, 11–20°	63	21
	Severe, ≥ 21°	7	2

HVA: Hallux valgus angle

IMA: Intermetatarsal angle

PPAA: Proximal phalangeal articular angle

**Figure 5:**
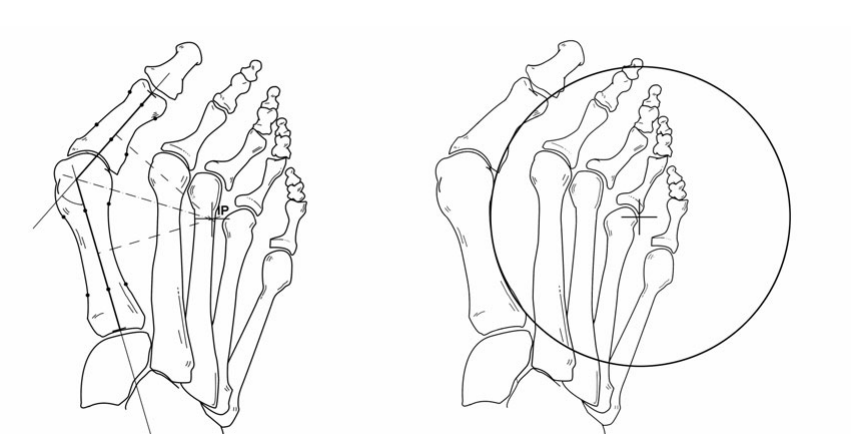
**Position of the intersection point (IP) (left) and of the center of the first metatarsophalangeal arc circumference (right) inside the foot in a case of severe hallux valgus deformity**.

**Figure 6:**
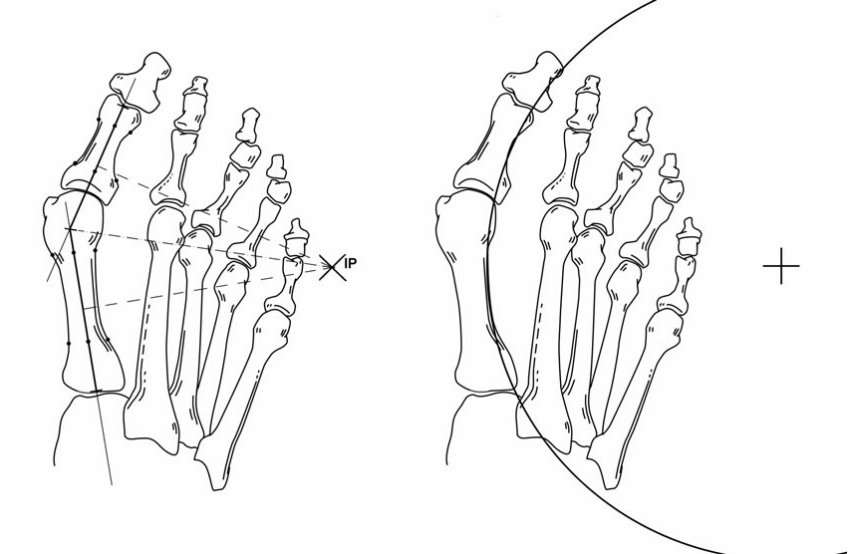
**Position of the intersection point (IP) and of the center of the first metatarsophalangeal arc circumference (right) out of the foot but within a distance of a foot width in a case of moderate hallux valgus deformity**.

The IP was found inside the foot in 111 (37%) patients and outside the foot in 190 (63.2%). The mean DL was 12.0 (7.2) mm. The mean length of the radius for the first metatarsophalangeal arc circumference was 13.0 (6.5) mm. Figures 5 and 6 show the categories established for the site of the IP and the location of the center of the circle inside the foot (Figure 5) or outside the foot (Figure 6).

In controls, the mean DL was 133.5 (50.2) mm (range 280-69.6) and the mean length of the radius for the first metatarsophalangeal arc circumference 150.7 (54.2) mm (range 274.7-65.6). Both the IP and the circle's center were located outside the foot and far away from a distance of a foot width.

The mean (SD) s-VAS score was 35.4 (20.0). As shown in Table 2, the three observers showed a high degree of agreement in the classification of the severity of hallux valgus deformity using VAS, with a correlation coefficient > 0.8 for all pair comparisons (*p *< 0.001). On the other hand, the intraclass correlation coefficient (ICC) was 0.846 (95% confidence interval [CI] 0.817–0.871) (*p *< 0.001). There were statistically significant differences between mean s-VAS scores according to severity of hallux valgus, that is, the s-VAS score was significantly higher when the IP was found inside the foot (52.8 [14.7]) as compared with outside the foot (25.3 [14.3]) (*p *< 0.001) (Table 2). On the other hand, the mean (SD) s-VAS score for the normal feet was 10.1 (8.2) for the first observer, 11.5 (10.9) for the second observed, and 9.4 (5.7) for the third observer.

**Table 2:** Severity of hallux valgus deformity and VAS scores for the three observers

Data	Mean (SD), mm	Pearson's r coefficient	p value
VAS scores			
Observer 1	32.0 (19.0)		
Observer 2	37.5 (19.9)		
Observer 3	36.8 (20.9)		
s-VAS*	35.4 (20.0)		
			
Agreement			
Observer 1 vs 2		0.873	< 0.001
Observer 1 vs 3		0.856	< 0.001
Observer 2 vs 3		0.876	< 0.01
s-VAS*			
			
Intersection point site			
Inside the foot	52.8 (14.7)		< 0.001^†^
Outside the foot	25.3 (14.3)		

VAS: Visual analogue scale

* Summary final VAS score obtained by factor analysis.

^† ^Pair comparisons of s-VAS: inside vs outside the foot.

With regard to mean HVA, IMA and PPAA values and severity of hallux valgus, measurements of all angles except PPAA showed significantly lower values when the IP was located out of the foot more distantly and vice versa, significantly higher values for severe deformities in which the IP was found inside the foot (*p *< 0.001) (Table 3).

**Table 3:** Mean (SD) angular values according to position of the intersection point

	Site of the intersection point		p value
Variables	Inside the foot	Outside the foot	Inside vs outside the foot
HVA	42.5 (7.5)	23.1 (8.8)	< 0.001
IMA	15.1 (3.3)	10.7 (3.0)	< 0.001
PPAA	7.4 (5.6)	7.6 (4.5)	0.802

HVA: Hallux valgus angle

IMA: Intermetatarsal angle

PPAA: Proximal phalangeal articular angle

Table 4 shows the correlation between s-VAS (severity of hallux valgus according to the observers) and the remaining measures. HVA and IMA showed a strong and significant correlation with s-VAS, that is, the greater hallux valgus deformity rated by the observers, the greater the values of these angles. On the other hand, severity of hallux valgus rated by the observers correlated negatively with DL, that is, the greater hallux valgus deformity rated by the observers, the shorter the DL.

**Table 4:** Correlation between summary VAS score and angular values and distance length (DL)

Data	Pearson's correlation coefficient	p value
HVA	0.857	< 0.001
IMA	0.823	< 0.001
PPAA	0.579	< 0.001
Distance length	-0.694	< 0.001

HVA: Hallux valgus angle

IMA: Intermetatarsal angle

PPAA: Proximal phalangeal articular angle

The relationship between each angle and DL is summarized in Table 5. In the univariate analysis, for each 10 mm of DL increase, the HVA decreases 0.8°, IMA decreases 0.5°, and PPAA increases 0.01°. In the multivariate analysis, an increase of 10 mm of DL causes decreases of 0.531° of HVA and 0.07° of IMA, and an increases of 0.04° of PPAA of the remaining angles for each case are maintaned unchanged. The application of these data to a theoretical model shows that for the IP to be found outside the foot, the mean values (SD) of angular measurements would be 40.46 (10.9)° for HVA, 14.26 (3.8)° for IMA, 36.04 (11.6)° and 7.53 (4.2)° for PPAA.

**Table 5:** Relationship between angular measurements (HVA, IMA and PPAA) and Distance Length

	Univariate			Multivariate		
	Correlation index	Beta coefficient	p value	R	Beta coefficient	p value
HVA	-0.805	-1.412	< 0.001	0.870	-0.531	< 0.001
IMA	-0.523	-0.278	< 0.001	0.425	-0.074	0.064
PPAA	0.015	0.010	0.795	0.151	0.044	0.484

HVA: Hallux valgus angle

IMA: Intermetatarsal angle

PPAA: Proximal phalangeal articular angle

**Figure 7:**
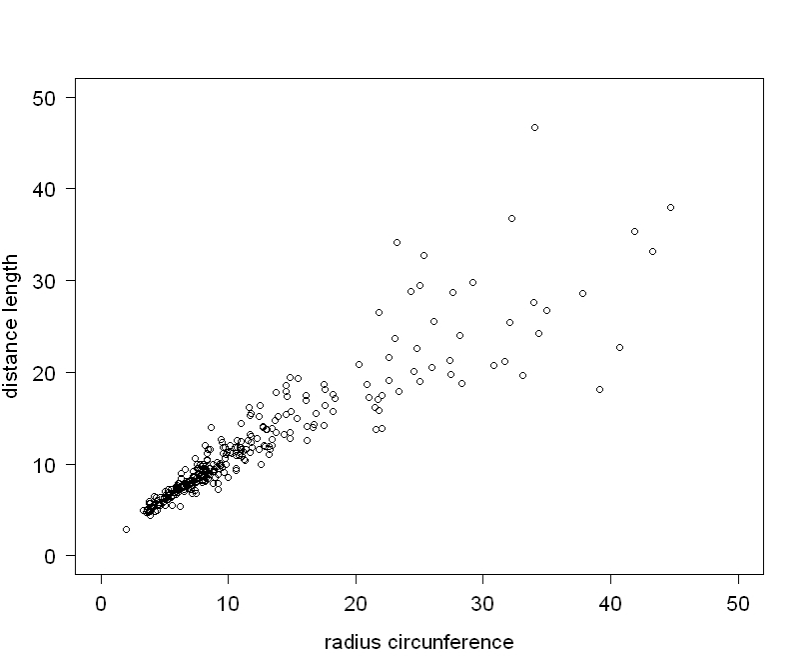
**Correlation between DL and radius of the first metatarsophalangeal arc circumference**.

Finally, an excellent correlation between DL and length of the radius of the circle that included the first metatarsophalangeal arc circumference was observed (*r *= 0.911, 95% CI 0.890–0.928, *p *< 0.001) (Figure 7).

## Discussion

The IP was located within the bony foot in all cases of severe hallux valgus and in some cases of moderate deformity whereas the point was observed out of the foot in the remaining cases. Measurements of all angles (HVA, IMA) except PPAA showed significantly higher values when the IP was located out of the foot more distantly and vice versa, significantly higher values in cases of severe deformities in which the IP was found inside the foot.

In controls, the mean values of HVA, IMA, and PPAA were within normal ranges, the VAS scores of the three independent observers were very low, with a mean of 10.4 (7), and both the IP and the center of the first metatarsophalangeal arch circumference were located far outside the foot and far away from a distance of a foot width.

On the other hand, the clinical assessment of hallux valgus deformity according to VAS scores given by independent observers on visual inspection of forefoot photographs was shown to be a reliable procedure. Garrow et al. [21] developed the Manchester scale, a clinical tool consisting of photographs of feet with fours levels of hallux valgus: none, mild, moderate, and severe. Both intratester and intertester realiability of grading hallux valgus using this approach have been found to be excellent, with kappa values of 0.77 and 0.86, respectively, suggesting that it is a useful tool for clinical and research purposes. Menz and Munteanu [22] determined the validity of this tool by correlating Manchester scale with hallux valgus measurements obtained from radiographs from 95 subjects (31 men and 64 women; mean age 78.6 [6.5] years). This study showed that the Manchester scale was highly correlated with HVA (Spearman's *ρ *= 0.73, *P *< 0.01) and moderately associated with IMA (*ρ *= 0.49, *P *< 0.01) obtained from radiographs. Analysis of variance revealed significant differences in mean HVA and IMA between the four Manchester scale categories. At the time of the study design, radiographic validation of the Manchester scale was still unpublished; for this reason, hallux valgus deformity was assessed using photographs by means of VAS.

In our study, the three observers showed a high degree of agreement with a correlation coefficient > 0.8 for all pair comparisons and an ICC of 0.846. The s-VAS score was significantly higher when the IP was found inside the foot as compared with outside the foot. In addition, HVA and IMA values showed a strong and significant correlation with s-VAS, that is, the greater deformity rated by the observers, the greater the values of these angles. On the other hand, severity of hallux valgus rated by the observers and by radiographic measures correlated inversely with DL, that is, the greater hallux valgus deformity rated by the observers and radiographic measures, the shorter the DL.

According to our data, if the external border of the fifth metatarsal is considered as the limit for the IP, in a case of severe hallux valgus, the mean values of the angles obtained were 42.5° for HVA and 15.1° for IMA, which are consistent with values of HVA ≥ 40° generally considered to define severe hallux valgus [8,14-17,32-34].

In a previous study, the location of the center of the circle formed by the first metatarsophalangeal arc circumference correlated significantly with HVA, DMMA, and IMA measurements [29]. In the present study, an excellent correlation between DL and length of the radius of the circle was found, so that, the position of the IP may have practical relevance for research purposes, since these lines can be easily drawn on weightbearing radiographs. Moreover, severity levels according to its position inside or outside the foot were significantly related not only to angular measurements, but also to clinical assessment of severity of bunion deformity according to VAS for visual inspection of photographs by three independent observers.

## Conclusion

The IP is a useful indicator of hallux valgus severity because showed a significant correlation with angular measurements, visual inspection of the degree of deformity and location of the center of the first metatarsophalangeal arch circumference. Geometric-based analysis offers a novel approach for further research in the field of hallux valgus and to explore different ways to consider hallux valgus deformity.

## Abbreviations

ANOVA: Analysis of variance; CI: Confidence interval; DL: Distance length; DMAA: Distal metatarsal angle; HVA: Hallux valgus angle; CC: Intraclass correlation coefficient; IMA: First intermetatarsal angle; IP: Intersection point of the perpendicular bisectors of the longitudinal axes of the first metatarsal and proximal phalanx; PPAA: Proximal phalangeal articular angle; SAS: Statistical analysis system; SD: Standard deviation; VAS: Visual analogue scale.

## Competing interests

The authors declare that they have no competing interests.

## Authors' contributions

CPV designed the study, interpreted the results, wrote the paper and prepared the final draft.

JV contributed to the study design, analyzed the data, interpreted the results and approved the final draft.
